# Assessing Community Pharmacists’ Management of Acute Diarrhea in Saudi Arabia: A Simulated Patient Study

**DOI:** 10.3390/healthcare12232385

**Published:** 2024-11-27

**Authors:** Faris S. Alnezary, Dina A. Alahmadi, Fatima N. Abduljaleel, Riham G. Alharbi, Fahad Alzahrani, Masaad S. Almutairi

**Affiliations:** 1 Department of Pharmacy Practice, College of Pharmacy, Taibah University, Madinah 41477, Saudi Arabia; fnezary@taibahu.edu.sa (F.S.A.); dina.alahmadi20@gmail.com (D.A.A.); fatimanmaj@gmail.com (F.N.A.); rihamalbadrani@gmail.com (R.G.A.); fzahrani@taibahu.edu.sa (F.A.); 2 Department of Pharmacy Practice, College of Pharmacy, Qassim University, Buraydah 51452, Saudi Arabia

**Keywords:** diarrhea, community pharmacists, simulated patient, antibiotic resistance, antibiotics

## Abstract

Introduction: Antimicrobial resistance poses a significant threat to global health, and community pharmacists are positioned to play a crucial role in mitigating this issue. The present study aimed to evaluate the extent of compliance among community pharmacists in Saudi Arabia with relevant regulations and clinical guidelines in the management of suspected infectious diarrhea. Method: This cross-sectional study employed simulated patients presenting with diarrhea to assess the management practices within 200 community pharmacies in two major cities across Saudi Arabia. Trained pharmacy students presented pharmacists with three case scenarios involving adult patients with diarrhea. Statistical analysis included descriptive statistics and chi-square tests to examine the relationships between pharmacist characteristics and practice categories. Results: The findings of this study indicate that the performance of community pharmacists in managing diarrhea is suboptimal. Notably, less adequate practice emerged as the predominant outcome at 63% (n = 126). Only 14% (n = 28) of pharmacists demonstrated adequate practice, while 23% (n = 46) exhibited poor investigative practice. Metronidazole dispensing increased across scenarios, from 16.92% (n = 11) in Scenario 1 to 30.3% (n = 20) in Scenario 3. Most pharmacists inquired about the patient’s age (72%; n = 144); however, only a limited number probed for symptoms of dehydration (5.5%, n = 11) and medication history (3%, n = 6). A significant association was found between geographical location and practice performance (*p* = 0.015). Conclusions: This study reveals significant deficiencies in the management of infection-related diarrhea, underscoring the urgent need for enhanced training and regulatory measures within community pharmacy settings in Saudi Arabia to improve patient care and effectively address antimicrobial resistance (AMR).

## 1. Introduction

Antibiotics serve as fundamental components of contemporary medical science, representing the primary and most effective line of defense against pathogenic microorganisms. However, their efficacy is increasingly jeopardized by the rise of antimicrobial resistance (AMR), predominantly attributable to misuse and overuse [1,2,3]. Among the numerous risks associated with this phenomenon is the significant threat to public health, as AMR contributes to increased morbidity and mortality, in addition to incurring substantial economic costs [4].

Recent reports indicate that AMR currently results in over 700,000 fatalities annually on a global scale, with projections suggesting a potential increase to more than 10 million lives lost by the year 2050 [5]. Collectively, these findings underscore the urgent necessity for more judicious use of antibiotics and the implementation of enhanced strategies for managing resistance.

If left unchecked, the economic threat posed by AMR could have catastrophic implications for the global economy, with projected losses reaching as high as USD 100 trillion [6]. Community pharmacists have been identified as crucial allies in combating the dangers associated with AMR [7]. Their presence is significant at the local level, and their extended hours of operation position them as key partners with the greatest access to healthcare services [8]. Pharmacies occupy a pivotal position at the intersection of patients and healthcare systems, where pharmacists function as gatekeepers in the dispensing of antibiotics, in alignment with the World Health Organization’s (WHO) 2015 global action plan on antimicrobial resistance [9,10]. This role is especially critical in community settings, where much of the misuse and overuse of antibiotics originates.

Diarrhea represents a prevalent issue within community pharmacies globally [11]. Although it was considered a minor condition in 2015, recent evidence indicates that it remains a significant public health concern, contributing to an estimated 1.31 million deaths across all age groups [12,13]. The etiology of diarrhea is extensive, encompassing a range of viral pathogens, including norovirus and rotavirus, as well as bacterial agents such as *Salmonella*, *Shigella*, and *Escherichia coli*, in addition to *Clostridioides difficile* [14]. In clinical practice, diarrhea is characterized by the passage of three or more loose or watery stools within a 24 h period, frequently accompanied by an increased frequency of bowel movements, abdominal pain or cramping, nausea or vomiting, and fever [15]. Prolonged or severe diarrhea poses the risk of dehydration, which may manifest through clinical signs such as dry mouth and tongue, muscle cramps, sunken eyes, reduced urine output, and general weakness [16]. These potential risks underscore the importance of implementing effective control measures and ensuring prompt management of diarrheal cases.

Under typical circumstances, the majority of diarrhea cases are characterized as mild and self-limiting; individuals are likely to seek advice or over-the-counter (OTC) treatments at a pharmacy as their initial recourse [17]. However, these cases present a significant public health risk and may result in complications or mortality, particularly among vulnerable populations such as young children, elderly, and immunocompromised individuals. To mitigate these risks, community pharmacists are expected to adopt an evidence-based approach that encompasses a two-step patient evaluation leading to appropriate interventions [18,19]. This process should take into account various factors, including the patient’s age, the duration and severity of symptoms, the presence of associated symptoms, recent dietary modifications, travel history, or current medication usage, with specific attention to antibiotics or anti-diarrheal agents [19]. This thorough patient history enables pharmacists to make informed decisions regarding patient care and, when necessary, to make appropriate referrals.

The guidelines established by the World Health Organization (WHO) for the management of infectious diarrhea are extensive and detailed [19]. The principal elements of diarrhea management include symptomatic relief, rehydration, and when deemed appropriate, antibiotic therapy [19,20]. Hydration, particularly through the use of oral rehydration salts (ORS), is acknowledged as the primary intervention in the treatment of diarrhea [21]. For symptomatic relief, the WHO recommends loperamide as the first-line therapy for adults experiencing diarrheal disease due to its efficacy and minimal central nervous system-related side effects [19]. Additionally, loperamide is classified as an OTC medication in numerous countries, thereby facilitating self-medication when necessary [22]. However, it is imperative to recognize that while symptomatic treatment may provide some relief, accurate identification and management of the underlying cause remain essential, particularly in cases of severe or persistent diarrhea.

In response to the emergence of AMR as a global priority for action, the Ministry of Health (MOH) in Saudi Arabia has implemented several strategic initiatives. Among these, the MOH developed a national antimicrobial stewardship plan aimed at achieving specific outcomes, including the rational use of antimicrobials, increased rates of treatment success, and a reduction in the development of AMR [23]. This initiative was further supported by a revised enforcement act in 2018, which established regulations prohibiting community pharmacies from dispensing antibiotics without a valid medical prescription [24]. The regulation imposes heavy fines on violators, imposing penalties up to SAR 100,000 (equivalent to USD ~26.6), cancellation/revocation of licenses, and imprisonment for not exceeding six months for violating pharmacists and pharmacies [25]. Notwithstanding these regulatory measures, instances of inappropriate antibiotic dispensing continue to occur in Saudi Arabia, exacerbating the growing AMR crisis [24,26]. Therefore, this study aims to assess community pharmacists’ compliance with established standards. Specifically, we conducted an evaluation analyzing pharmacists’ responses to a simulated patient presenting with cases indicative of infectious diarrhea. The findings of this study will contribute to a deeper understanding of antimicrobial dispensing practices and their potential correlation with the progression of antibiotic resistance profiles in Saudi Arabia.

## 2. Materials and Methods

### 2.1. Study Design

The study utilized a cross-sectional, observational design and applied a simulated patient (SP) approach. This technique is well-established for evaluating the competencies, practices, and quality of counseling of community pharmacists (CPs) in real-world settings [27]. Specifically, the study employed a covert simulated patient methodology, similar to the method used by Hamadouk et al. [28].

### 2.2. Sampling Method and Size

The study encompassed community pharmacies located in the cities of Madinah and Al-Khobar, including both chain and independent establishments. To ensure adequate geographical representation of community pharmacies in both cities, a simple random sampling technique was employed. Utilizing the Raosoft sample size calculator, it was determined that, assuming a population of 400 community pharmacies, a 95% confidence level, a margin of error of ±5%, and an expected response distribution of 50%, the minimum required sample size is n = 197 (Raosoft sample size calculator (2004); available online at: http://www.raosoft.com/samplesize.html (accessed 2 February 2024)). To enhance statistical power, the sample size was increased to a total of 200 pharmacies. Consequently, the margin of error at a 95% confidence level is adjusted to approximately 4.8%, as per the Raosoft calculator. This approach has resulted in a heightened level of confidence and a more representative sample.

### 2.3. Simulated Patients

A group of three pharmacy students from Taibah University’s College of Pharmacy were trained as simulated patients (SPs) to visit community pharmacies in the cities of Madinah and Al Khobar. Each participant completed 66 visits, with 22 visits allocated to each of the three scenarios, totaling 198 visits. To reach the target sample size of 200, two additional visits were conducted. The study commenced in March 2023 and extended over a period of three months.

In order to minimize any potential differences in the presentation of the SP, several standardization measures were implemented. Each scenario was delivered by the same SP per that scenario to minimize inter-presenter variability. All SPs were similar in age (22–24 years) and background, pharmacy students, and they underwent training to standardize their presentation of cases through both role-plays and a comprehensive review of the scenario. Moreover, feedback sessions were conducted at regular intervals to ensure similar scenarios were delivered during the study duration.

Three theoretical patient scenarios (Table 1) regarding adult diarrhea were developed based on a comprehensive examination of the reference guide “Symptoms in the Pharmacy” [29]. These scenarios were further enriched with relevant evaluation points and questions, drawing from the available research on essential factors to consider when triaging and advising patients with diarrhea [20,30,31].

### 2.4. Data Collection Form

To mitigate biases arising from inadequate information, each visit was carefully documented and subsequently transcribed onto a designated form. The form was divided into eight sections: (i) pharmacy information (location, type, and visit time); (ii) pharmacist information (nationality, gender, and estimated age); (iii) history taking; (iv) dispensed treatment(s); (v) recommended actions and advice on treatments; (vi) instructions regarding dispensed medication; (vii) advice on food and fluid intake; and (viii) additional information, including the pharmacist’s willingness to provide counseling. The assessment of pharmacists’ attitudes and willingness to counsel patients was adapted from the existing literature [32,33] and is detailed in the outcome measurement section.

### 2.5. Data Collection Procedures

The study team evaluated the completed forms as part of the feedback process and to ensure clarity and comprehensiveness. To reduce inter-rater variability, all encounters for a specific case scenario were conducted by the same simulated patient. Visits were scheduled at various times of day over three months, including both work and holiday periods, to mitigate potential fluctuations in pharmacist availability and to prevent detection. To maintain independence of observations, each pharmacy was visited only once across the three case scenarios, and only one pharmacist was assessed per pharmacy.

### 2.6. Outcomes

#### 2.6.1. Primary Outcome

This study aims to evaluate the performance of community pharmacists in Saudi Arabia by assessing specific fundamental competencies that contribute to a comprehensive understanding of their practice. These competencies involve gathering essential information from the simulated patient, assessing the case, and utilizing expertise to decide on appropriate interventions for different adult diarrhea scenarios.

##### Primary Outcome Measurement

The primary outcome was measured using a developed rating scale for each scenario, based on established standards from the reference guide “Symptoms in the Pharmacy” [29]. The scales assess three competencies: patient assessment, intervention, and pharmacist professional behavior.

The measure utilizes a 3-Point Likert Scale, with responses ranging from 0.0 to 2.0. The average total points for each statement and visit were classified into three intervals: adequate practice (2.0–1.4), less adequate practice (1.3–0.7), and poor practice (0.6–0.0) [34].

Patient assessment skill was evaluated based on inquiries about the patient’s identity, age, and diarrhea characteristics (duration, severity, dehydration, and accompanying symptoms) across all scenarios. Scenario-specific questions were included to assess the underlying cause of diarrhea.

The intervention competency was evaluated differently for each scenario. For the first scenario (traveler’s diarrhea), the assessment focused on the correct dispensing of first-choice therapy, including dosage, frequency, and duration [35]. For the second and third scenarios, the evaluation centered on referral advice and guidance on food and fluid intake [30]. Additionally, the dispensing of antibiotics without a prescription was assessed in all scenarios.

Professional behavior was subjectively evaluated based on the use of open/closed-ended questions, active listening skills, explanation detail, professional demeanor, and voice tone, which was in line with previous publications [30,36]. Active listening was assessed through observations of eye contact, verbal acknowledgments, and clarifying questions. Professional demeanor was primarily exemplified by the pharmacist’s patience, while voice tone was evaluated for audibility, clarity, pace, and warmth.

#### 2.6.2. Secondary Outcomes

The secondary study outcomes included (1) the frequency of antibiotics dispensing without a prescription; (2) an identification of variations in dispensing practices among chain pharmacies, independent community pharmacies, and other types of pharmacies; and (3) an analysis of the determinants influencing pharmacy and pharmacist characteristics on primary outcomes

### 2.7. Data Analysis

We employed descriptive statistics to present the data, reporting categorical variables as frequencies and percentages. The Pearson Chi-Square test was utilized to evaluate the associations between the characteristics of pharmacists and their respective practice categories. A significant level was established at a *p*-value of less than 0. Statistical analyses were conducted using Stata version 16.1 (Stata Corp., College Station, TX, USA).

## 3. Results

Table 2 presents the demographic and practice performance of community pharmacists in Madinah, Saudi Arabia (n = 83) and Al Khobar, KSA (n = 761). A total of 200 pharmacists participated in the study, with the majority (n = 135; 67.5%) hailing from the Madinah region. The sample comprised (n = 129, 64.5%) chain pharmacies and (n = 71, 35.5%) independent pharmacies. Among the pharmacy types, Chain 2 exhibited the highest representation (n = 55; 27.5%), followed by independent pharmacies (n = 71; 35.5%). Chain 4 demonstrated the lowest representation (n = 8; 4%). Significant differences in practice performance were observed among the various pharmacy types (*p* = 0.002): the highest proportion of less adequate practice was noted in Chain 3 (n = 16; 80.0%), whereas a higher prevalence of poor practice was recorded in Chain 4 (n = 5; 62.5%).

The majority of the pharmacists were non-Saudi (n = 171, 85.5%) and predominantly male (n = 191, 95.5%). The age distribution of the participating pharmacists revealed that most were aged between 30 and <40 years (n = 90; 45%), followed by those aged >20 to ≤29 years (n = 46; 24.5%). No significant associations were found between nationality (*p* = 0.828), gender (*p* = 0.750), and age groups (*p* = 0.432) with practice performance.

The majority of pharmacy visits occurred during evening hours (5 pm to 8 pm), with a total of n = 87 (43.5%), followed by night visits (after 8 pm), totaling n = 56 (28%). The timing of visits did not significantly influence practice performance (*p* = 0.358). The number of years in service demonstrated a statistically significant relationship with practice performance (*p* < 0.001). A substantial majority of interactions (n = 182; 91%) lasted less than two minutes. Conversations of longer duration (≥2 min) were more likely to be categorized as adequate practice, with 50% (n = 9 out of 18) classified as such and none classified as poor practice.

Overall, a significant proportion of pharmacists exhibited less adequate practice (n = 126; 63%), while a minority demonstrated adequate practice (n = 28; 14%). A significant effect of geographical location on practice was identified (*p* = 0.015), with the distribution showing 15 (23.1%) in Al Khobar exhibiting adequate practices compared to 13 (9.6%) in Madinah.

Table 3 presents a comprehensive analysis of the assessment questions posed by community pharmacists when patients present with diarrhea-related complaints. The frequency of inquiries regarding patient assessment elements varied across different categories of questions. The most frequently asked question pertained to the age of the patient, with 72% (n = 144) of pharmacists addressing this aspect. Similarly, 48% (n = 96) of pharmacists inquired about additional symptoms accompanying diarrhea, such as fever or abdominal cramps. In contrast, questions regarding the duration and severity of diarrhea were less frequently posed, with only 20.5% (n = 41) and 17.5% (n = 35) of pharmacists assessing these factors, respectively. Patient identity was questioned by 15.5% (n = 31) of pharmacists, while recent dietary changes were discussed by a smaller proportion of 14.5% (n = 29) of pharmacists.

The data also indicate several areas where pharmacist inquiries were notably infrequent. Signs and symptoms of dehydration were explored by only 5.5% of the surveyed pharmacists (n = 11), and inquiries regarding potential causative factors, such as irritable bowel syndrome, were similarly rare. Furthermore, only 3% (n = 16) of pharmacists asked about previous medications, excluding antibiotics. Questions concerning medications attempted for relief were noted at a meager 4% (n = 8), while inquiries related to recent antibiotic use or current antibiotics being administered accounted for merely 1.5% each (n = 3). Additionally, questions pertaining to recent travel abroad were infrequently posed, with only one pharmacist (0.5%) inquiring whether other household members had experienced similar symptoms and no pharmacists asked about prior episodes of diarrhea preceding the current episode.

Table 4 presents a comprehensive analysis of community pharmacists’ practice performance across three distinct diarrhea scenarios. In Scenario 1, which pertains to potential traveler’s diarrhea, data from 65 respondents indicate that a significant majority of pharmacists demonstrated less adequate practice, with 52 individuals (80%) falling into this category. A minimal proportion, specifically 9.23% (n = 6), exhibited adequate practice, while 10.77% (n = 7) were classified as demonstrating poor practice. In Scenario 2, which addresses the treatment of antibiotic-associated diarrhea, a total of 69 participants were evaluated. The percentage of pharmacists demonstrating less adequate practice decreased to 60.87% (n = 42) in comparison to the findings from the first scenario. However, the incidence of poor practice rose to 30.43% (n = 21), while the rate of adequate practice remained constant at 8.7% (n = 6). Scenario 3, which illustrates cases of food poisoning (n = 66), involved a different distribution of practice performances among the three scenarios examined. Here, the incidence of adequate practice increased significantly to 24.24% (n = 16), the highest observed among all scenarios. Conversely, the proportion of pharmacists exhibiting less adequate practice decreased to 48.48% (n = 32), while the rate of poor practice remained relatively high at 27.27% (n = 18).

Table 5 presents a detailed analysis of community pharmacists’ management approaches to the three diarrhea scenarios. In the case of traveler’s diarrhea (n = 65), pharmacists exclusively dispensed medication in Scenario 1, with a substantial majority of approximately (95.38%, n = 62) opting for this course of action. This scenario represented the highest level of medication dispensing among the evaluated cases. One pharmacist (1.54%, n = 1) chose to refer the patient without dispensing medication, while two pharmacists (3.07%, n = 2) both provided medication and a referral. Notably, none of the pharmacists decided to take no action in this scenario. There was a noticeable increase in patient referrals, with 10.14% (n = 7) of pharmacists opting to refer the patient without dispensing medication. Three pharmacists (4.3%, n = 3) both dispensed medication and referred the patient, while one pharmacist (1.45%, n = 1) took no action. In contrast, in Scenario 2, representing antibiotic-associated diarrhea (n = 69), a minor adjustment in medication dispensing was observed. The majority of pharmacists (n = 58) still favored dispensing medication, constituting approximately 84.05%. Pharmacists assisted 10.14% (n = 7) of patients who opted for medication based on referral, with no medications dispensed to them. In terms of dispensing medication and referring patients, three pharmacists (4.3%) engaged in both actions, while one pharmacist took no action (1.45%, n = 1). Furthermore, medication dispensing declined to 78.7% (n = 52) in Scenario 3, which involved cases of food poisoning (n = 66), representing a marked decrease compared to the previous scenarios. The highest proportion of pharmacists referring patients without medication occurred in this scenario, with 12.12% (n = 8) opting for this approach. A total of five pharmacists (7.57%) engaged in both the dispensing of medication and the referral of the patient, while one pharmacist (1.52%, n = 1) did not take any action.

When analyzing all outcomes collectively, the data exhibit a trend of decreased medication dispensing coupled with an increase in patient referrals from Scenario 1 to Scenario 3. This decline ranged from 95.38% (n = 62) of pharmacists who exclusively dispensed medication in Scenario 1 to a reduced percentage in subsequent scenarios. Conversely, the proportion of pharmacists who exclusively referred patients increased from 1.54% (n = 1) in Scenario 1 to 10.14% (n = 7) in Scenario 2 and further escalated in the third Scenario, reaching 12.12% (n = 8). Interestingly, medication dispensing and patient referral were most common in Scenario 3 (7.57%, n = 5), followed by Scenario 2 (4.3%, n = 3), and least common in Scenario 1 (3.07%, n = 2).

Table 6 presents a detailed analysis of all dispensed antibiotics utilized by community pharmacists across the three diarrhea scenarios. In Scenario 1, which pertains to traveler’s diarrhea (n = 65), metronidazole emerged as the most frequently dispensed antibiotic 16.92% (n = 11). The second most dispensed antibiotic, nifuroxazide, was dispensed by 4.62% (n = 3) of pharmacists. Both tinidazole and a combination of metronidazole with diloxanide furoate were dispensed by 1.54% of pharmacists (n = 1 for each), while secnidazole was prescribed by only 3.08% (n = 2). Notably, ciprofloxacin was not dispensed in this scenario.

In Scenario 2, which addresses antibiotic-associated diarrhea (n = 69), there was an increase in the dispensing of metronidazole, with 21.74% (n = 15) of pharmacists selecting this option. Once again, nifuroxazide ranked as the second most preferred choice, and it was selected by 5.8% of pharmacists (n = 4). One pharmacist dispensed tinidazole, and the combination of metronidazole and diloxanide furoate was dispensed by 1.45% (n = 1) each. Importantly, no pharmacists dispensed secnidazole or ciprofloxacin in this scenario.

Conversely, in Scenario 3, which illustrates a case of food poisoning (n = 66), the dispensing of metronidazole reached the highest level across all scenarios, accounting for 30.3% (n = 20). Notably, 3.03% of pharmacists (n = 2) dispensed ciprofloxacin in this case, marking its first instance of prescription in the preceding scenarios.

## 4. Discussion

This study offers a comprehensive examination of the management of acute diarrhea by community pharmacists in Saudi Arabia and highlights practice deficiencies that closely align with findings from other researchers. Adequate practice was demonstrated by 14% (n = 28) of pharmacists; however, the majority exhibited less adequate practice (63%, n = 126) or poor performance (23%, n = 46). These findings are consistent with studies conducted in Pakistan, Iraq, and Turkey, which also reported inadequate management of diarrhea cases by community pharmacists [37,38,39].

### 4.1. History-Taking Practices

Inadequate history-taking practices have emerged consistently in our study as well as in other studies. The inquiry regarding the patient’s age was the most prevalent across all studies, with a frequency ranging from 72% in our study to 98.3% in Gondar, Ethiopia [40]. However, significant components of the patients’ medical histories were often overlooked. In our study, only 20.5% of pharmacists inquired about the duration of diarrhea, which is notably lower than that reported in Baghdad (78.7%) and Gondar (76.7%) [38,40]. This consistent deficiency in comprehensive history-taking across various countries underscores the pressing global need for enhanced education for general practitioners regarding this fundamental aspect of patient care.

### 4.2. Medication Dispensing Patterns

Across all trials, high rates of drug dispensing were observed, ranging from 77.1% in Pakistan to approximately 100% in Germany [37,41]. However, the class of medications selected varied by country. In Saudi Arabia, metronidazole exhibited the highest distribution frequency, accounting for 16.92% in Scenario 1, with prevalence increasing to a maximum rate of 30.3% in Scenario 3. One of the contributing factors for high metronidazole dispensing in our study could be a widespread misconception among healthcare providers—including pharmacists—that metronidazole acts more as an intestinal sanitizer than an antibiotic. Comments made by some of the community pharmacists during the simulated patient visits indicating that metronidazole acts as an intestinal ‘cleanser’ reflect this perception. This non-prescriptive belief may perhaps result in its indiscriminate use even though it is an antibiotic and should, therefore, be given when clinically indicated. Such misunderstanding, as this finding demonstrates, should be addressed through educational interventions to avoid inappropriate antimicrobial stewardship. 

Conversely, emerging data from other nations indicate differing patterns of drug prescriptions; for instance, ciprofloxacin was predominant in Gondar at 52.6%, loperamide was prevalent in Germany at 88%, and antiamoebic agents were reported at 58.7% in Pakistan [37,40,41]. These disparities are likely attributable to local treatment guidelines, the availability of pharmaceuticals, and cultural preferences.

### 4.3. Antibiotic Stewardship Challenges in Saudi Pharmacists

Prior to 2018, a significant number of pharmacists in Saudi Arabia dispensed antibiotics without prescriptions. According to Alshreedy et al. (2020), before the enactment of relevant legislation, 70.7% of pharmacists participated in this practice. Following the implementation of the law, this figure experienced a substantial decline, with the rates falling to 12.1% for pharyngitis and 5.2% for urinary tract infections (UTIs) [42]. However, a recent study conducted during the COVID-19 pandemic revealed that over 15% of pharmacists continued to dispense antibiotics without prescriptions [43]. In our study, it was observed that 30.5% of pharmacists dispensed antibiotics without obtaining a prescription.

This practice violates Saudi Pharmacy Law and Saudi FDA regulations, which leads to monetary fines and may suspension of the pharmacy license itself [44]. In spite of these policies, arbitrary antibiotic dispensing still occurs due to various factors such as patient pressure for immediate treatment, cultural beliefs on the effectiveness of antibiotics, and economic incentives for pharmacy [45]. These statistics are notably higher than findings from previous studies, suggesting potential deficiencies in current monitoring efforts and a deviation from established treatment recommendations for diarrhea [19].

The role of community pharmacists is key to the promotion and provision of rational use of antibiotics, making their contribution imperative in combatting the growing threat posed by antimicrobial resistance. Although they are supposed to follow currently existing guidelines based on evidence and should be a part of the national AMR plan in Saudi Arabia, our results suggest that there is still an unmet need for better interventions. Addressing these alarmingly high rates of over-the-counter antibiotic dispensing requires interventions in policy, such as electronic monitoring systems to assess antibiotic dispensing in real time, compulsory reporting, and regular audits on pharmacy employees, with significant non-compliance consequences. Finally, we recommend that continuing education programs be required to incorporate the evidence related to the management of infectious diarrhea, non-pharmacological treatment options for otherwise self-limiting conditions, and identification of red flags requiring referral. The implementation of these interventions, combined with standard treatment protocols for infectious disease syndromes commonly managed in community pharmacies, may contribute to the alignment of pharmacy practice with national antimicrobial stewardship aims.

### 4.4. Oral Rehydration Solution (ORS) Dispensing

A concerning finding across all studies is the significant underutilization of oral rehydration solution ORS. In our study, it was not identified as the primary treatment of choice in any scenario. Furthermore, studies conducted in various countries revealed that the prevalence of ORS was alarmingly low, with rates of 0% in Germany and as high as 35.5% in Quetta City [41,46]. ORS is the first-line treatment recommended by the World Health Organization [19]. ORS therapy prevents dehydration, re-establishes electrolyte homeostasis, and decreases both the severity and duration of diarrheal episodes [47]. Evidence indicates that this intervention has contributed substantially to the global reduction in diarrhea-related mortality [48]. The very low dispensing rate of ORS (15.5%) identified by us represents a deviation from clearly documented practice guidelines and best practices around diarrhea management.

There are various reasons for such underutilization of ORS in a systematic manner. Our first finding is that only 5.5% (n = 11) of pharmacists assessed dehydration symptoms, which indicates a greater knowledge gap for pharmacists who manage patients with diarrhea than our findings of rehydration being the key main role in dealing with diarrhea. Second, commercial considerations such as the higher profit margins of antibiotics and anti-diarrheal drugs compared to ORS may also affect pharmacists’ choices. Third, cultural norms and the desire of patients for “stronger” medicines may reinforce the idea that ORS alone is inadequate therapy [49].

This persistent trend of systematic underuse of ORS across diverse geographical contexts highlights a critical gap in the management of diarrhea, especially in light of recent guidelines that advocate for the use of ORS as a frontline treatment [15,19,30].

### 4.5. Patient Referral Practices

In all scenarios evaluated, a discernible trend of increasing patient referrals was observed, with the lowest referral rate occurring in Scenario 1 (1.54%) and the highest in Scenario 3 (12.12%). This finding aligns with the limited referral rates reported in two separate studies: 5% in Gondar and an even lower rate of 2.7% in Baghdad [38,40]. In contrast, the study conducted in Khartoum presented a more nuanced perspective, revealing that 17% of pharmacists recommended medical consultation when presented with a case lacking additional information, which increased to 42.6% when provided with further explanation [28]. These results underscore a global necessity to enhance pharmacists’ ability to recognize and refer cases of higher acuity.

### 4.6. Practice Adequacy and Counseling

The performance of pharmacists in practice has been found to be less than satisfactory, with 63% of assessments categorized as less adequate and 23% classified as poor. While various studies employed different categorization methods, they consistently reported suboptimal practices. For instance, a study conducted in Turkey indicated that counseling practices were generally inadequate, with a notable number of patients lacking sufficient education regarding medication usage, including aspects of storage and duration [39].

### 4.7. Duration of Interaction

The findings of our study indicate that 91% of interactions lasted less than two minutes. Notably, longer exchanges (defined as two minutes or more) were associated with higher quality practice; specifically, 50.0% (n = 9 out of n = 18) of conversations that lasted two minutes or longer were deemed adequate, with no instances of poor practice reported. Indeed, this association between the quality and duration of community pharmacist consultation demonstrates that sufficient time for patient assessment should normally take at least 3–4 min if adequate counseling with patients is to occur [50]. A similar trend of short consulting times was observed in Quetta City, where the average duration was recorded at 2.37 min [46]. Short patient encounters lead to inadequate assessment of patients and insufficient counseling, which deteriorates the pharmaceutical care process. Oh et al. (2002) have also shown that longer consultation times were required for medication-related problems [50]. Their research indicated that just a simple patient contact took an estimated average of three minutes.

To address this time constraint while maintaining pharmacy workflow efficiency, several practical solutions warrant consideration. Changing pharmacy counter designs to include private consultation areas, investments in queue managing systems, and delegating non-clinical responsibilities to pharmacy technicians [51,52,53]. Over time, systemic alterations such as pharmacist-to-support-staff ratios, systems of appointment-based consultations for certain conditions, and standardized assessment protocols to maximize time could contribute to these desired outcomes [51,54,55].

The consistent evidence across multiple studies demonstrating that patient contact time is insufficient in terms of quality of care highlights a significant area for improvement. The evidence suggests that prolonged consultation periods are essential for community pharmacists to have adequate time to conduct meaningful interactions with patients.

### 4.8. Strengths and Limitations

Several characteristics enhance the validity and relevance of this research. The implementation of simulated patient methodology provided a robust framework for assessment across various scenarios, effectively mitigating potential biases associated with self-reporting practices among pharmacists. The inclusion of three distinct adult diarrhea case vignettes, coupled with chart abstraction conducted by a single reviewer, not only broadened the scope of assessment but also minimized interrater variability. A significant strength of this study is that all interactions were overseen by certified pharmacists, in contrast to studies conducted in Pakistan and Quetta City, where a minimal proportion of cases were resolved by pharmacists [37,46]. This disparity underscores critical differences in regulatory practices and professional engagement between countries, which may be indicative of the quality of care delivered. Furthermore, the study’s execution in two different cities, catering to populations with diverse sociocultural characteristics, enhances the generalizability of the findings concerning adherence to enforcement laws in Saudi Arabia.

However, it is important to acknowledge certain limitations. The subjectivity involved in grading professionalism may have introduced a source of bias into the results. The absence of feedback regarding performance constituted a missed opportunity for improvement, depriving pharmacists of immediate access to performance-related insights. Moreover, while the influence of pharmacist demographics on practice patterns is well-documented, our simulated patient methodology precluded the collection of detailed demographic profiles of non-Saudi pharmacists, as this would have compromised the covert nature of observations and introduced bias. The study did not adequately identify potential distractions that could have impeded effective communication between pharmacists and patients, nor did it address issues related to privacy, which may have obstructed conversations. Additionally, the study design did not facilitate the assessment of behavioral changes over time; thus, it does not provide insight into the sustainability of any potential interventions or practice changes. Furthermore, our pharmacy selection process focused solely on geographical representation across districts. Further studies will benefit from sampling around multiple stratification factors (e.g., parts of the city close to healthcare facilities and commercial centers, etc.) and demographic variables across locations. These considerations could shed light on how pharmacy placement and infrastructure around pharmacies may impact dispensing.

## 5. Conclusions

A significant disparity has been identified between the management of infectious diarrhea by community pharmacists and the established ideal practice in Saudi Arabia, with only a minority (14%) of knowledge and practice scores deemed adequate. These findings underscore three critical areas for development: the implementation of targeted education programs for pharmacists, the enhancement of regulatory oversight concerning antibiotic dispensing, and the necessity for region-specific interventions aimed at improving adherence to evidence-based guidelines. These steps are essential for advancing patient care and addressing the challenge of antimicrobial resistance within the context of community pharmacy.

## Figures and Tables

**Table 1:** Simulated patient scenarios.

Scenarios	Traveler’s Diarrhea	Antibiotic-Associated Diarrhea	Food Poisoning
**For who?**	Housekeeper	Sister	Me
**Age (years)**	25	21	23
**Duration**	2 days	2 days	2 days
**Frequency**	2–3/day	4/day	4/day
**Other Symptoms**	Abdominal cramps, increased thirst, watery stool	Low-grade fever (37.9 °C, abdominal pain, pale skin, fatigue, dizziness	Fever, nausea, vomiting
**Family Members**	No	No	No
**Tried Medications**	No	No	Nothing was tried except for salt in water
**Antibiotics**	No	Ciprofloxacin	No
**Change in Food**	No	No	Yes
**Recent Travel**	Returned from Indonesia (1 week ago)	No	No
**Angle**	History taking (Recent travel)	History taking (current antibiotic use) and knowledge (loperamide contraindications)	History taking (Recent change in food and long period of s/s)

**Table 2:** Demographic characteristics and practice performance of community pharmacists in Madinah and Al Khobar, Saudi Arabia.

Variable	Number	Percent	Practice Performance			
			Adequate Practice (n = 28, 14%)	Less Adequate Practice (n = 126, 63%)	Poor Practice (n = 46, 23%)	*p*-Value
**Type of Pharmacy**						
Chain 1	46	23.00%	10 (22.22%)	23 (51.11%)	12 (26.67%)	**0.002**
Chain 2	55	27.64%	10 (18.52%)	39 (72.22)	5 (9.26%)	
Chain 3	20	10.05%	2 (10%)	16 (80%)	2 (10%)	
Chain 4	8	4.02%	0	3 (33.33%)	6 (66.66%)	
Independent	71	35.68%	6 (8.33%)	45 (62.50%)	21 (29.17%)	
**Geographical location**						
Madinah	135	67.50%	13 (9.63%)	93 (68.89%)	29 (21.48)	**0.015**
Al Khobar	65	32.50%	15 (23.08%)	33 (50.77%)	17 (26.15%)	
**Time of the Visit**						
Morning (8 am–11 am)	18	9.00%	2 (11.11%)	12 (66.67%)	4 (22.22%)	0.358
Afternoon (12 pm–4 pm)	39	19.50%	5 (12.82%)	30 (76.92%)	4 (10.26%)	
Evening (5 pm–8 pm)	87	43.50%	14 (16.09%)	48 (55.17%)	25 (28.74%)	
Night (After 8 pm)	56	28.00%	7 (12.50%)	36 (64.29%)	13 (23.21%)	
**Duration of Service (min)**						
<2	182	91.00%	19 (10.61%)	114 (63.69%)	46 (25.70%)	**<0.001**
≥2	18	9.00%	9 (42.86%)	12 (57.14%)	0	
**Nationality**						
Saudi	29	14.25%	5 (17.24%)	17 (58.62%)	7 (24.14%)	0.828
Non-Saudi	171	85.50%	23 (13.45%)	109 (63.74%)	39 (22.81)	
**Estimated Age of Pharmacist**						
20–29	49	24.50%	10 (20.41%)	25 (51.02%)	14 (28.57%)	0.432
30–39	90	45.00%	14 (15.22%)	58 (63.04%)	20 (21.74%)	
40–49	46	23.00%	3 (6.82%)	33 (75%)	8 (18.18%)	
50–59	10	5.00%	1 (10%)	7 (70%)	2 (20%)	
>60	5	2.50%	28 (14%)	126 (63%)	46 (23%)	
**Gender**						
Female	9	4.50%	1 (11.11%)	5 (55.56%)	3 (33.33%)	0.750
Male	191	95.50%	27 (14.14%)	121 (63.35%)	43 (22.51%)	

**Table 3:** Assessment questions for community pharmacist visits.

Pharmacist’s Assessment Items	Number of Pharmacists	Percent
Patient identity	31	15.50%
Age	144	72.00%
Duration of diarrhea	41	20.50%
Severity of diarrhea	35	17.50%
Dehydration symptoms (i.e., fatigue, dry mouth)	11	5.50%
Other symptoms (i.e., fever, abdominal cramps)	96	48.00%
Affected family members	1	0.50%
Recent travel abroad	3	1.50%
Change in food habit	29	14.50%
Other possible causative factors (e.g., IBS)	11	5.50%
Recent diarrhea	0	0.00%
Medication history (other than antibiotics)	6	3.00%
Current or recent administration of antibiotic	3	1.50%
Medicines tried to relieve the diarrhea	8	4.00%

**Table 4:** Practice performance of community pharmacists across three diarrhea scenarios.

	Adequate Practice	Less Adequate Practice	Poor Practice
**Scenario 1**	6 (9.23%)	52 (80%)	7 (10.77%)
**Scenario 2**	6 (8.7%)	42 (60.87%)	21 (30.43%)
**Scenario 3**	16 (24.24%)	32 (48.48%)	18 (27.27%)

**Table 5:** Community pharmacists’ management approaches to three distinct diarrhea scenarios.

	Medication Dispensed Only	Patient Referred Only	Medication Dispensed and Patient Referred	None
**Scenario 1**	62 (95.38%)	1 (1.54%)	2 (3.07%)	0
**Scenario 2**	58 (84.05%)	7 (10.14%)	3 (4.3%)	1 (1.45%)
**Scenario 3**	52 (78.7%)	8 (12.12%)	5 (7.57%)	1 (1.52%)

**Table 6:** Distribution of antibiotics dispensed by community pharmacists across three diarrhea scenarios.

	Metronidazole	Nifuroxazide	Tinidazole	Secnidazole	Ciprofloxacin	Metronidazole + Diloxanide Furoate
**Scenario 1**	11 (16.92%)	3 (4.62%)	1 (1.54%)	2 (3.08%)	0	1 (1.54%)
**Scenario 2**	15 (21.74%)	4 (5.8%)	1 (1.45%)	0	0	1 (1.45%)
**Scenario 3**	20 (30.3%)	0	0	0	2 (3.03%)	0

## Data Availability

The original contributions presented in this study are included in the article. Further inquiries can be directed to the corresponding author.
